# Nickel-Atom Doping as a Potential Means to Enhance the Photoluminescence Performance of Carbon Dots

**DOI:** 10.3390/molecules28145526

**Published:** 2023-07-20

**Authors:** Wenqi Kong, Can Li, Zhongqi Sun, Fucheng Gao, Jinfan Zheng, Yanyan Jiang

**Affiliations:** 1 Key Laboratory for Liquid-Solid Structural Evolution and Processing of Materials, Ministry of Education, Shandong University, Jinan 250061, China; 15045116075@163.com (W.K.); cancan626@163.com (C.L.); sunzq529@163.com (Z.S.); 2 Department of Neurology, Qilu Hospital, Shandong University, Jinan 250100, China

**Keywords:** carbon dots, nickel-atoms doping, fluorescence imaging, anticounterfeiting

## Abstract

Heteroatom doping, particularly with nonmetallic atoms such as N, P, and S, has proven to be an effective strategy for modulating the fluorescent properties of carbon dots (CDs). However, there are few reports on the regulation of the photoluminescence of CDs by transition-metal doping. In this work, nickel-doped CDs (Ni-CDs) were fabricated using the hydrothermal approach. Ni atoms were incorporated into the sp^2^ domains of the CDs through Ni-N bonds, resulting in an increased degree of graphitization of the Ni-CDs. Additionally, Ni-atom doping served to shorten the electron transition and recombination lifetimes, and suppress the nonradiative recombination process, resulting in an absolute fluorescence quantum yield of 54.7% for the Ni-CDs. Meanwhile, the as-prepared Ni-CDs exhibited excellent biocompatibility and were utilized for fluorescent bioimaging of HeLa cells. Subsequently, the Ni-CDs were employed as fluorescent anticounterfeiting inks for the successful encryption of two-dimensional barcodes. Our work demonstrates a novel heteroatom doping strategy for the synthesis of highly fluorescence-emitting CDs.

## 1. Introduction

Carbon dots (CDs), zero-dimensional carbon-based nanomaterials with characteristic dimensions below 10 nm, possess intrinsically tunable photoluminescence (PL) [1,2]. Their unique fluorescence (FL) properties have enabled widespread applications in bioimaging [3], FL anticounterfeiting [4], information encryption [5], and sensor detection [6,7]. The FL properties of CDs can be attributed to emissions from carbon core states, surface states, and molecular states [8,9]. However, a deeper understanding of the FL mechanism of CDs remains elusive. Meanwhile, most CDs exhibit low absolute FL quantum yields (FLQY) [10], which represents a major impediment to their commercialization. Hence, developing CDs with high FLQY and elucidating their emission mechanisms have become the focus of fundamental CD research.

Nonmetallic atom doping with N, P, and S has been proven to be an effective method for regulating the PL properties of CDs [11,12]. For example, Gong et al. prepared B- and N-codoped CDs, which endowed CDs with a FLQY of 28% and successfully achieved lysosome targeting [13]. In another study, Bi et al. prepared F- and N-doped CDs, and under optimal excitation, the absolute FLQY of F- and N-doped CDs reached as high as 18.25%. Essentially, the strategy of doping heteroatoms is still achieved through the regulation of the carbon source [14]. Therefore, the selection of a carbon source is crucial for preparing high-performance FL-emitting CDs.

Metal ions, especially heavy metal ions, often induce FL quenching of CDs [15,16,17]. Therefore, it is traditionally believed that the addition of heavy metal ions is not conducive to the FL emission of CDs. However, recent studies have shown that transition metal atom (TMA)-doped CDs prepared from transition metal ion complexes as carbon precursors have significantly improved FL emission properties due to the incorporation of TMAs [18]. For example, Chen et al. successfully prepared Mn-doped CDs, and the results showed that the bridging effect of Mn-O bonds induced a redshift in Mn-CD fluorescence emission with an absolute quantum yield of 70.12% [19]. Subsequently, Jiang et al. also confirmed that Mn-atom doping can enhance the PL ability of CDs [20]. However, there are still few reports on the in-depth study of the effect of TMA doping on the fluorescence emission mechanism of CDs.

**Scheme 1:**
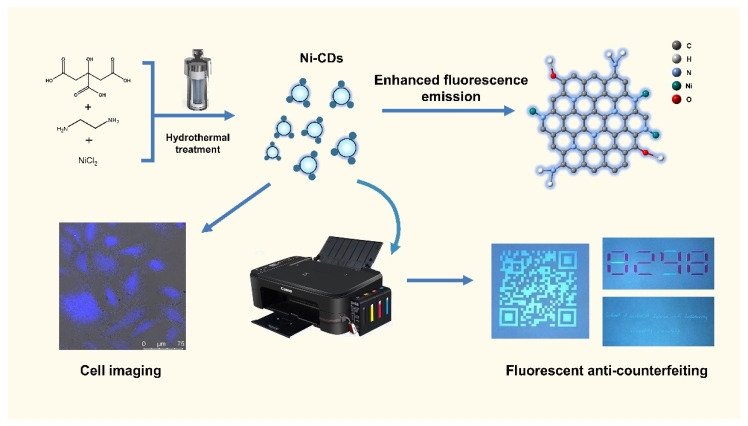
The schematic preparation process procedures for Ni-CDs and the development of cell imaging and fluorescent anticounterfeiting.

In this work, we prepared Ni-doped CDs (Ni-CDs) via a one-step hydrothermal method using citric acid (CA) and ethylenediamine (EDA) as carbon sources and NiCl_2_ as the metal source. EDA can anchor Ni atoms through amino groups. The results showed that Ni atoms existed in CDs in the form of Ni-N bonds. The absolute fluorescence quantum yield of Ni-CDs was 54.7%, which was 14% higher than that of pure CDs (P-CDs). Systematic characterization showed that Ni doping expanded the sp^2^ domain of CDs, increased the number of photo-generated charges, accelerated the recombination rate of carriers, suppressed the nonradiative transition process of excited electrons, and ultimately improved the FLQY of CDs. Based on the excellent FL emission performance of Ni-CDs, in vitro cell imaging of Hela cells and fluorescence anticounterfeiting were achieved (Scheme 1). This work provides a new idea for preparing high-performance FL-emitting CDs.

## 2. Results and Discussion

**Figure 1:**
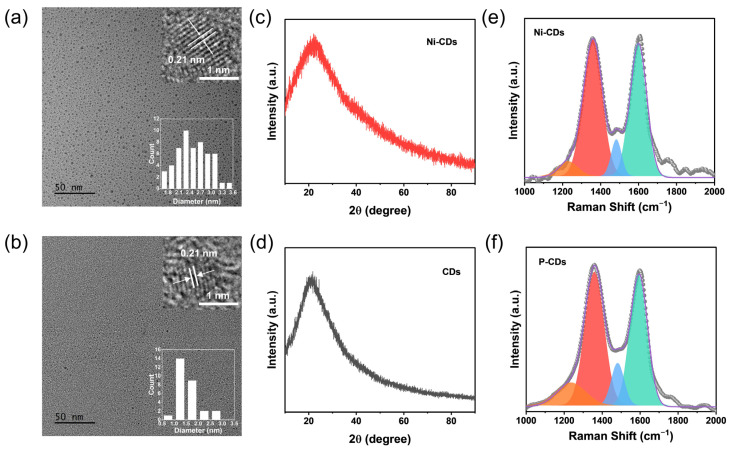
TEM image, HR-TEM image, and histogram of the particle size distribution of (**a**) Ni-CDs and (**b**) P-CDs. The X-ray diffraction patterns of (**c**) Ni-CDs and (**d**) P-CDs. The Raman spectrum of (**e**) Ni-CDs and (**f**) P-CDs.

The nickel-doped CDs (Ni-CDs) were prepared via a facile hydrothermal approach at 180 °C for 10 h. CA and EDA served as the carbon precursors and NiCl_2_ as the metal source, with EDA acting as a chelating ligand to complex Ni^2+^ ions via amine groups (-NH_2_) [21]. The pristine CDs (P-CDs) were prepared analogously without adding Ni^2+^ ions. As evidenced by transmission electron microscope (TEM) images (Figure 1a,b), Ni-CDs exhibited an average diameter of 2.4 nm, exceeding that of P-CDs (1.5 nm). They both showed a lattice fringe spacing of 0.21 nm, concordant with the (100) plane of graphite [22]. As illustrated in Figure 1c,d, Ni-CDs and P-CDs displayed a prominent broad peak at 22.3° in X-ray diffraction patterns, attributable to graphitic carbon [23,24]. In addition, the Raman spectrum of Ni-CDs (Figure 1e) exhibited four peaks. The peaks at 1230 and 1487 cm^−1^ are attributed to sp^3^-C of the carbon core or surface groups. The peaks 1361 and 1560 cm^−1^ corresponded to the sp^2^-C hybridization [25]. The A_sp3_/A_sp2_ ratio was determined to be 1.25 for Ni-CDs, lower than that of 1.44 for P-CDs (Figure 1f), indicating the enhanced graphitization of P-CDs upon Ni doping [26], as supported by the TEM observations.

**Figure 2:**
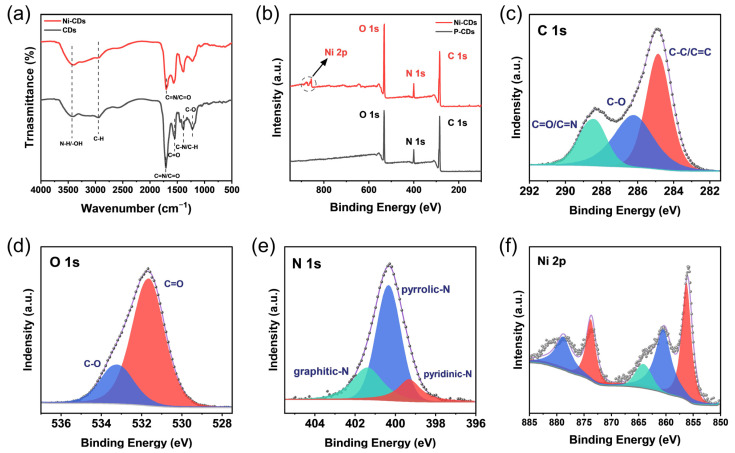
(**a**) The FT-IR spectra of Ni-CDs and PCDs. (**b**) The full XPS spectra of Ni-CDs and P-CDs. The high-resolution XPS spectra of Ni-CDs, including (**c**) C 1s, (**d**) O 1s, (**e**) N 1s, and (**f**) Ni 2p.

The surface chemical properties of Ni-CDs were further probed using Fourier transform infrared (FT-IR) spectra and X-ray photoelectron spectra (XPS). In the FT-IR spectrum (Figure 2a), the P-CDs showed absorption peaks at 3411.97 and 2941.56 cm^−1^, which correspond to N-H/O-H and C-H groups [27], respectively; the band at 1711.83 cm^−1^ is attributed to the absorption of C=N/C=O, and the band at 1550.76 cm^−1^ to C=O absorption [28]. The band at 1382.03 cm^−1^ originates from asymmetric C-N/C-H stretching, and the band at 1220.96 cm^−1^ originates from C-O stretching [26]. Moreover, the peak for C=N/C=O shifts from 1712 to 1696 cm^−1^ after Ni doping due to Ni chelation with N atoms, implying the formation of Ni-N bonds [29]. 

The full XPS survey spectra showed the presence of C, O, N, and Ni in Ni-CDs at 61.85%, 28.81%, 7.3%, and 2.04% atomic percentage, respectively (Figure 2b, red line). In contrast, the P-CDs contained only C (67.74%), O (24.21%), and N (8.05%) (Figure 2b, black line). The high-resolution C 1s spectrum of Ni-CDs (Figure 2c) exhibited three peaks at 284.8, 285.7, and 288.1 eV, corresponding to C-C/C=C, C-N, and C=O/C=N bonds, respectively [30,31]. The O 1s spectra (Figure 2d) showed peaks at 531.7 eV (C=O) and 533.2 eV (C-O). The high-resolution N 1s spectrum of Ni-CDs (Figure 2e) revealed three components assigned to graphitic-N, pyrrolic-N, and pyridinic-N [32]. Notably, the binding energy (BE) of pyridinic-N appeared at 398.6 eV in P-CDs (Figure S1) but shifted to a higher BE (399.2 eV) in Ni-CDs, indicating Ni coordination with N atoms [33]. The Ni 2p spectra (Figure 2f) showed peaks at 856.1 eV (Ni 2p3/2) and 873.8 eV (Ni 2p1/2), confirming the presence of Ni atoms in Ni-CDs [34]. The satellite peak at 864.2 eV suggests trace amounts of NiO, probably due to the exposure of Ni-CDs in the air [26]. Considering that divalent Ni^2+^ ions were incorporated into the CDs via the formation of Ni-EDA complexes during synthesis, we postulated that Ni^2+^ ions were effectively doped into the CDs through the establishment of Ni-N bonds [26,34].

**Figure 3:**
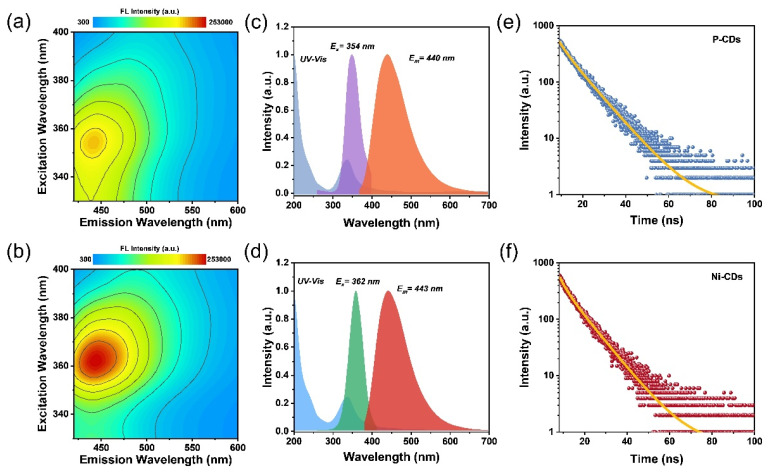
The 3D fluorescence topographical map of (**a**) P-CDs and (**b**) Ni-CDs. The UV-vis, PL excitation, and emission spectra of (**c**) P-CDs and (**d**) Ni-CDs. The PL decay curves of (**e**) P-CDs and (**f**) Ni-CDs.

The PL characteristics of both Ni-CDs and P-CDs are presented in Figure 3. Remarkably, the three-dimensional (3D) FL spectra of Ni-CDs and P-CDs exhibit a similar emission peak centered at approximately 440 nm. Moreover, under a variety of excitation wavelengths, the fluorescence emission profiles of Ni-CDs and P-CDs remain similar. In particular, with excitation wavelengths ranging from 300 to 360 nm (Figures S2 and S3), the emission wavelength remains unchanged, which can be attributed to the molecular state emission of CDs [35]. In contrast, when the excitation wavelengths span 370–400 nm, a redshift in the emission wavelength is observed, shifting from 454 to 488 nm, ascribed to surface-state FL emission [36,37]. The absolute FLQY of P-CDs and Ni-CDs amounts to 40.7% and 54.7% (Figures S4 and S5), respectively, indicating that Ni atom doping enhances the FL emission ability of the CDs. 

The UV-vis absorption spectra of P-CDs and Ni-CDs exhibit pronounced absorption peaks at approximately 335 nm, attributed to the n-π* transition of surface functional groups possessing lone electron pairs [4]. Meanwhile, the PL excitation profiles of Ni-CDs and P-CDs display peaks at approximately 350 nm, approximating the absorption bands in lower energy regions, implying that their PL emission originates from absorption in lower energy areas. The PL decay of Ni-CDs and P-CDs is monitored at an excitation wavelength of 375 nm. The average PL lifetime, derived from double exponential fitting, amounts to 3.53 ns for Ni-CDs, surpassing the corresponding value of 2.87 ns for P-CDs.

**Figure 4:**
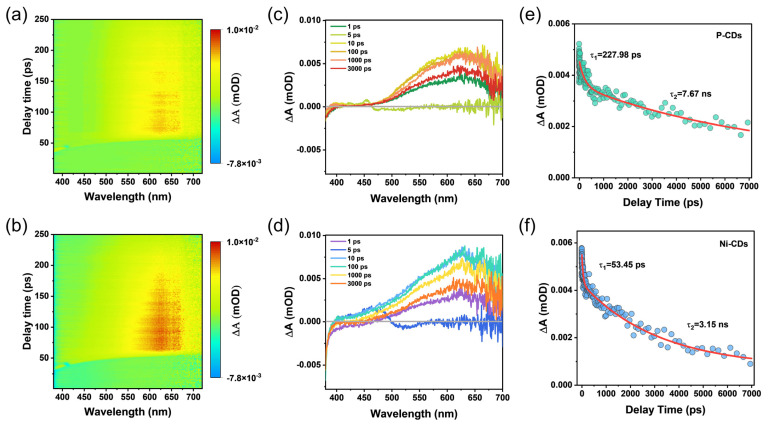
The pseudo-3D TA spectra of (**a**) P-CDs and (**b**) Ni-CDs. The TA spectra of (**c**) P-CDs and (**d**) Ni-CDs were recorded at specified delay times under 365 nm excitation. The TA kinetic traces of (**e**) P-CDs and (**f**) Ni-CDs at 365 nm within 7000 ps.

To further elucidate how Ni-atom doping affects the PL mechanism of CDs, we performed transient absorption (TA) spectroscopy measurements with a 365-nm pulsed laser under identical conditions. The pseudo-3D TA spectra (Figure 4a,b) revealed broad photoinduced absorption bands for both CDs in the 550–675 nm range. Ni-CDs showed a markedly stronger signal and a wider absorption band than P-CDs, indicating enhanced and faster stimulated emission. The positive absorption peaks of both CDs at 500–675 nm (Figure 4c,d) corresponded to the excited state absorption (ESA) of charge-separated states after photoexcitation, attributed to the overlapping electron-hole absorption [38]. Ni-CDs had a substantially higher initial absorption than P-CDs, suggesting that Ni doping generated more charges in CDs under 365 nm pump laser excitation. The ESA signal of Ni-CDs peaked at 100 ps and then gradually decreased with increasing delay time, possibly owing to the reduced ground state bleaching caused by the transition between valence band electrons and electron-poor trap states [39]. To further probe the charge transfer process, we monitored the photogenerated charge dynamics trajectories of Ni-CDs and P-CDs at 625 nm. Notably, both CDs exhibited decay curves that fit well with a double-exponential model (Figure 4e,f). The rapid decay of 300 ps in both curves was related to the direct recombination of photogenerated charges and charge transfer to capture levels, while the slow decay of several ns was attributed to the recombination of trapped charges [40]. Compared with P-CDs, Ni-CDs had shorter τ_1_ and τ_2_, indicating that Ni-N bonds facilitated electron transport by increasing the sp^2^ domain of CDs and accelerating the electron recombination rate [41]. In combination with the surface functional groups and TA spectral analysis results, we concluded that Ni doping increased the number of excited electrons in CDs under laser irradiation and enhanced electron transfer. Moreover, Ni-N bonds filled the vacancies in CDs, suppressing the nonradiative transition recombination process of CDs and ultimately improving their FLQY. Simultaneously, the existence of Ni-N bonds alters the energy level structure of CDs, consequently resulting in extended FL lifetimes for Ni-CDs.

**Figure 5:**
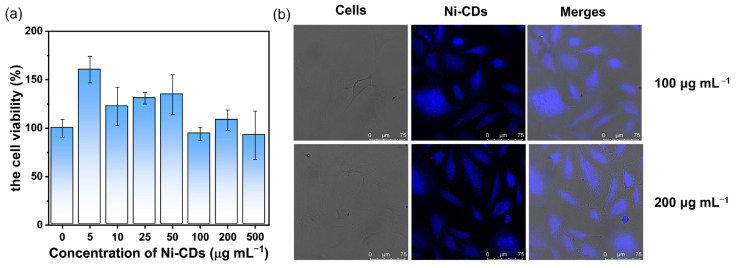
(**a**) The survival rate of Hela cells under different concentrations of Ni-CDs. (**b**) The CLSM images of HeLa cells treated with Ni-CDs for 4 h.

The biocompatibility of Ni-CDs was further investigated by using the 3-(4,5-dimethylthiazol-2-yl)-2,5-diphenyltetrazolium bromide (MTT) assay. The viability of Hela cells remained at 92.6% even though the concentration of Ni-CDs reached 500 μg/mL, affirming the splendid cytocompatibility of Ni-CDs [42] (Figure 5a). Subsequently, we investigated the potential of Ni-CDs as in vitro fluorescent imaging probes. As portrayed in Figure 5b, bright blue fluorescence emission was observed under a confocal fluorescence microscope after coincubating different concentrations of Ni-CDs (100 or 200 μg/mL) with Hela cells for 4 h, validating their good in vitro cell imaging performance [43,44,45].

**Figure 6:**
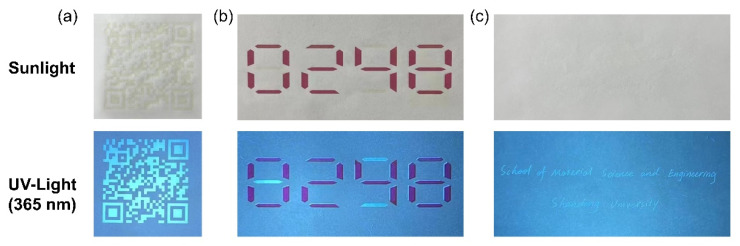
(**a**) Photographs of prints utilizing Ni-CDs as fluorescent ink under solar irradiation and 365 nm laser excitation. (**b**) Photographs of numeral information printed utilizing Ni-CDs as fluorescent ink under solar irradiation and 365 nm laser excitation. (**c**) Photographs of encrypted textual information utilizing Ni-CDs as fluorescent ink under solar irradiation and 365 nm laser excitation.

The Ni-CDs possess high FLQY, excellent chemical stability, and superb water solubility, making them highly promising for rapid and intelligent FL-based anticounterfeiting printing [46,47]. We utilized the aqueous solution of Ni-CDs (5 mg/mL) as fluorescent ink for experiments using a desktop inkjet printer. QR codes and numbers were printed on nonfluorescent commercial paper with the Ni-CDs ink, respectively. As illustrated in Figure 6a, under sunlight, the printed QR code exhibited lower resolution, resulting in its inability to be scanned successfully to obtain relevant information. However, upon irradiation with a 365 nm laser, the printed QR code emitted blue fluorescence, which enhanced its resolution and allowed successful scanning. We then explored the application of Ni-CDs in fluorescent digital anticounterfeiting. As shown in Figure 6b, under sunlight irradiation, incorrect number information (0248) was observed on paper. When irradiated with a 365 nm laser, the fluorescence emission of Ni-CDs revealed the concealed number information, thus obtaining the correct number information (8298). Moreover, the aqueous solution of Ni-CDs was added to a pen, and encrypted text was written on paper with nonfluorescent background. As depicted in Figure 6c, the information written on the paper was invisible under sunlight owing to its nearly colorless characteristic, whereas the encrypted information was clearly visible under 365 nm irradiation. Based on the Ni-CDs fluorescent ink, due to its green preparation, invisibility under sunlight, facile design, and excellent chemical stability, it will lead to developments in the field of optical anticounterfeiting printing.

## 3. Methods

### 3.1. Materials

CA, EDA, and NiCl_2_ were purchased from McLean. The 1000 Dalton cellulose dialysis bag was obtained from Beijing Solarbio Science & Technology Co., Ltd. (Beijing, China). 

### 3.2. Synthesis of Ni-CDs and P-CDs

CA (384 mg), EDA (50 μL), and nickel hexahydrate (170 mg) were dissolved in 15 mL of aqueous solution. The solution was placed in an autoclave and heated in an oven at 180 °C for 10 h. After cooling to room temperature, the solution was dialyzed through a dialysis membrane (500 Da) in ultrapure water for 6 h to remove unreacted substrates and small impurities. Finally, the brown powder was obtained after freeze-drying for 72 h and set aside. In addition, a group of nickel-free carbon dots was configured for control tests. (The P-CDs were otherwise identical to the method described above, except that no nickel was added).

### 3.3. Ink Printing Tests

It is important to note that the experiments were carried out using aqueous solutions of Ni-CDs. Anticounterfeit labels were prepared using Ni-CDs created according to the above method as fluorescent inks. The original ink in the printer cartridge was cleaned up, and the prepared Ni-CDs were added as ink. Handwritten images were obtained by adding the prepared Ni-CDs directly to the ink capsule of a pumping pen and then writing on paper. A commercial nonfluorescent paper was used for all papers in the experiments.

## 4. Conclusions

In summary, we successfully prepared Ni-doped carbon dots (Ni-CDs) that significantly improved the fluorescence quantum yield of CDs. Experimental results showed that the main reason for the increased fluorescence quantum yield was the presence of Ni atoms in the form of Ni-N bonds in the CDs, which expanded the sp^2^ domain of the CDs, increased the number of photogenerated charges, accelerated the rate of carrier recombination, and suppressed the nonradiative transition process of excited electrons. In addition, we also demonstrated that Ni-CDs exhibited excellent fluorescence chemical stability, good biocompatibility, and extremely strong water solubility, making them highly promising for applications in cell imaging in vitro and optical anticounterfeiting printing.

## Data Availability

The data presented in this study are available on request from the corresponding author. The data are not publicly available due to subsequent research on this topic is still ongoing.

## Supplementary Materials

The following supporting information can be downloaded at: https://www.mdpi.com/article/10.3390/molecules28145526/s1, Figure S1: The high-resolution N1s spectrum of P-CDs; Figure S2: The fluorescence emission spectra of P-CDs at different excitation wavelengths (from 300 to 400 nm); Figure S3: The fluorescence emission spectra of Ni-CDs at different excitation wavelengths (from 300 to 400 nm); Figure S4: The absolute fluorescence quantum yield of P-CDs; Figure S5: The absolute fluorescence quantum yield of Ni-CDs.
