# Supplementary material for: Nickel-Atom Doping as a Potential Means to Enhance the Photoluminescence Performance of Carbon Dots
Source: Molecules. 2023 Jul 20;28(14):5526. doi: 10.3390/molecules28145526 (PMC10386264; doi:10.3390/molecules28145526)
Supplement: Supplementary file 1 [file molecules-28-05526-s001.zip › molecules-2450979-supplementary.pdf]

Supporting Information

Nickel-Atoms Doping as a Potential Means to Enhance the Photoluminescence Performance of Carbon Dots

Wenqi Kong[1], Can Li[1], Zhongqi Sun[1], Fucheng Gao [1,*], Jinfan Zheng[2,*] and Yanyan Jiang [1,*]

Affiliations:

1. Key Laboratory for Liquid-Solid Structural Evolution and Processing of Materials, Ministry of Education, Shandong University, Jinan 250061, China
2. Department of Neurology, QiluHospital, Shandong University, Jinan 250100, China

Experimental Section

Material characterizations. The morphological information of Ni-CDs and P-CDs were obtained on transmission electron microscope (TEM; HT-7700, HITACHI and FEI Titan3 Cubed 60-300). Fourier transform infrared spectrometer (Nicolet 6700, Thermo Scientifuc) was used to analyze the surface functional groups of the sample. X-ray photoelectron spectroscopy (ESCALAB 250Xi, Thermo Electron, USA) was used to confirm the chemical state of Ni-CDs and P-CDs. The ultraviolet-visible spectrum was obtained from UV-visible spectrophotometer (SPECORD 200 PLUS, Jena, Germany). The printer model used for fluorescent anti-counterfeiting is Canon 3680. The scanning confocal fluorescence imaging microscope (CLSM800, Carl Zeiss, Germany) was applied to observe the cell imaging.

Determination of Hela cell viability:

In the first step of the experiment, Hela cells in the logarithmic growth phase of each experimental group were subjected to trypsin digestion, suspended in complete culture medium, and counted. The cell density for each group was then determined based on the rate of cell growth, with a set density of 2000 cells/well for this experiment, and this process was repeated three times. Once the cells had completely settled, their density was observed under a microscope to ensure consistency among the experimental groups. Subsequently, the Hela cells were cultured in a cell culture incubator for 24 hours, followed by the addition of Ni-CDs at various concentrations (0-500 μg/ml). After 20 hours, 20 μL of 5 mg/mL MTT was added to each well without changing the medium. Four hours later, the culture medium was completely removed, and 100 μL of DMSO was added to dissolve the formazan crystals. The plate was then shaken for 2-5 minutes, and the OD values of each experimental group were detected using an enzyme-linked immunosorbent assay reader at 490/570 nm. Finally, the survival rate of Hela cells in different groups was calculated based on the OD values obtained.

Cell Imaging Capacity Determination

The confocal laser scanning microscope (CLSM) was used to evaluate the cell imaging capacity of Ni-CDs. HeLa cells were seeded in culture dishes with a cell density of 5 × 10^3 per well, and cultured in a cell incubator with 5% carbon dioxide at 37 °C for 24 h. After complete adherence, the cells were treated with 100 or 200 μg mL^{-1} of Ni-CDs, incubated for 4 h, the culture medium was discarded, the cells were washed with PBS (pH 7.4) for 3 times to remove excess nanocomposite materials. After fixing with formaldehyde solution and washed carefully, the cells were placed under confocal microscope for fluorescence image observation. During the shooting process, the excitation wavelength range of the instrument was set between 390-410 nm, and the emission wavelength range was set between 430-460 nm.

Supplementary Figures

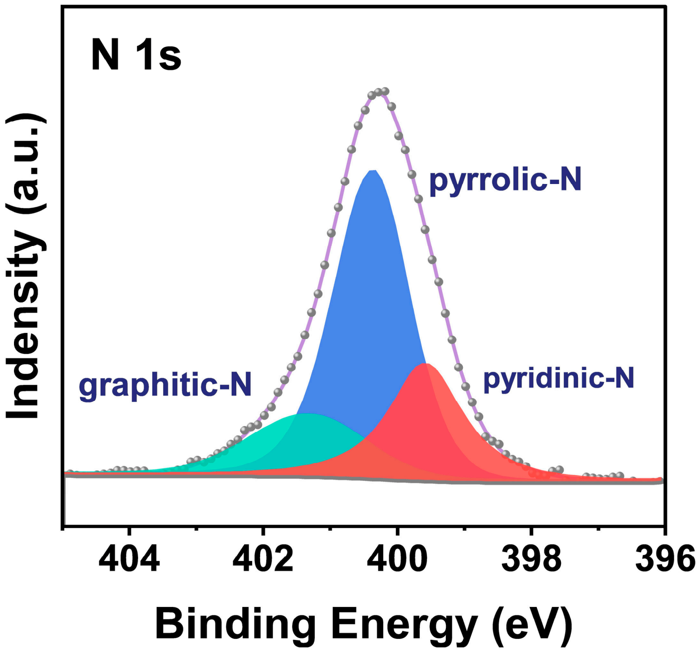

Figure S1. The high resolution N1s spectrum of P-CDs

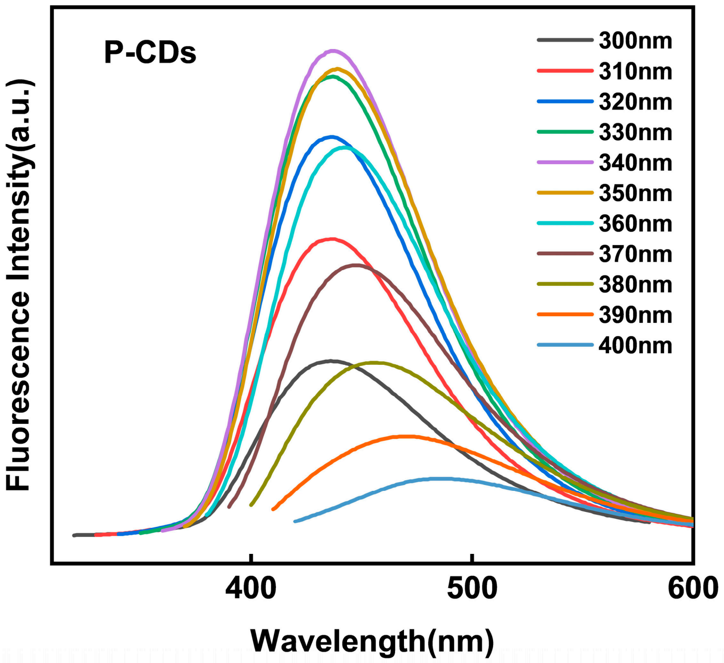

Figure S2. The fluorescence emission spectra of P-CDs at different excitation wavelengths (from 300-400 nm)

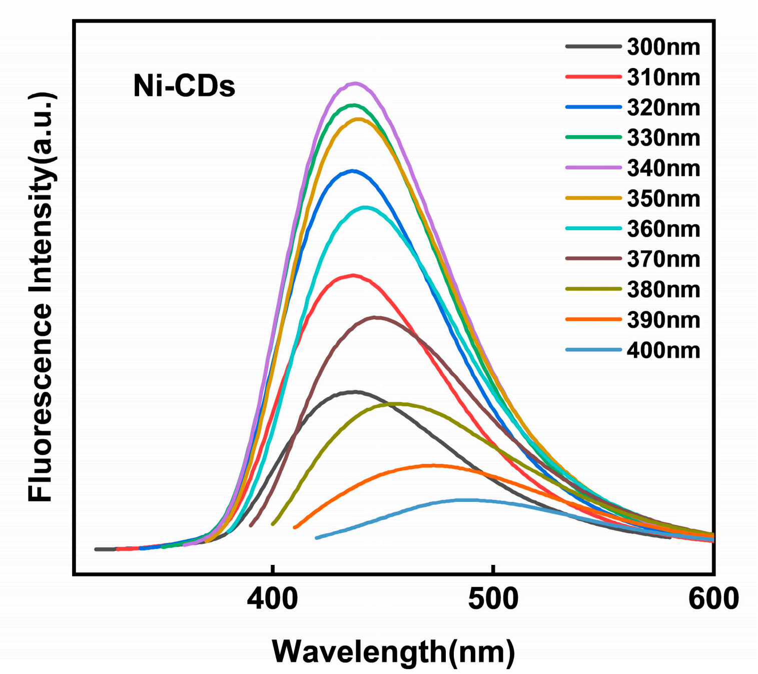

Figure S3. The fluorescence emission spectra of Ni-CDs at different excitation wavelengths (from 300-400 nm)

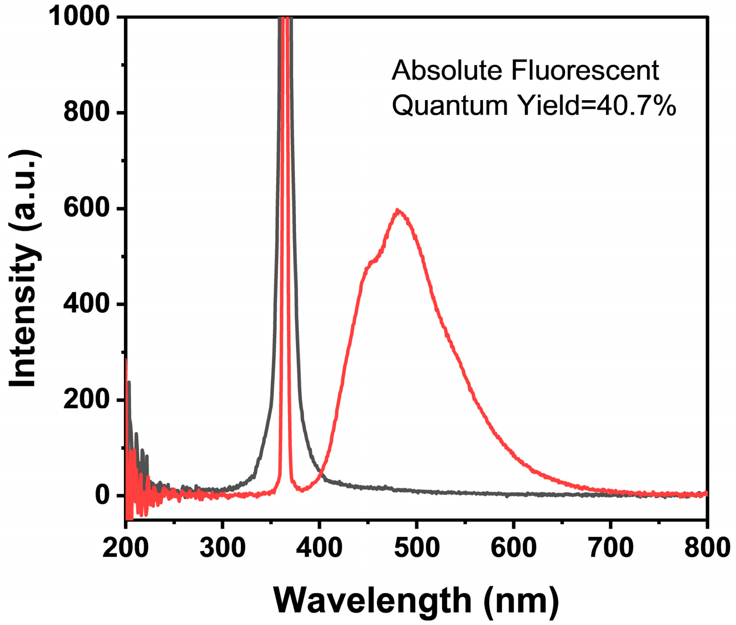

Figure S4. The absolute fluorescence quantum yield of P-CDs

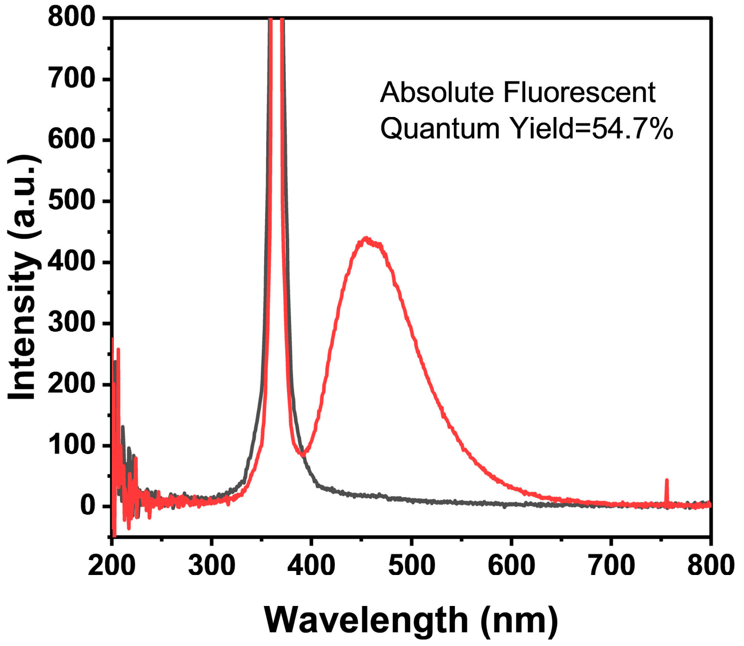

Figure S5. The absolute fluorescence quantum yield of Ni-CDs
